# Evaluation of date palm pollen (*Phoenix dactylifera* L.) encapsulation, impact on the nutritional and functional properties of fortified yoghurt

**DOI:** 10.1371/journal.pone.0222789

**Published:** 2019-10-15

**Authors:** Wedad Mohamed El-Kholy, Tarek Nour Soliman, Amira Muhammad Galal Darwish

**Affiliations:** 1 Department of Dairy Research, Food Technology Research Institute (A.R.C.), Alexandria, Egypt; 2 Department of Dairy, Food industries and nutrition Research Division, National Research Centre, Dokki, Cairo, Egypt; 3 Department of Food Technology, Arid Lands Cultivation Research Institute (ALCRI), City of Scientific Research and Technological Applications (SRTA-City), New Borg El-Arab, Alexandria, Egypt; Higher Institute of Applied Sciences and Technology of Gabes University of Gabes, TUNISIA

## Abstract

The aim of this study was to evaluate Egyptian date palm pollen (DPP) grains composition, physical and functional potentials in comparing with two forms; 80% ethanol extract, and nanoencapsulated form. Functional yoghurt fortified with DPP in three forms was prepared and their physicochemical, microstructure, texture and sensory characteristics were assessed. The micro morphology was explored via Scanning Electron Microscope (SEM). Fourier Transform Infrared (FTIR) spectroscopy was employed for functional groups detection. Phenolic compounds were detected by High Performance Liquid Chromatography (HPLC) while fatty acids were identified via Gas Liquid Chromatography (GLC). Cytotoxicity of DPP nanocapsules was evaluated against RPE1 cell line (BJ1). The Egyptian date palm pollen grains evaluation revealed its rich content of protein and carbohydrate (36.28 and 17.14 g/ 100g), high content of Fe, Zn and Mg (226.5, 124.4 and 318 mg/100g), unsaturated fatty acids ω-3, ω-6 and ω-9 (8.76, 20.26 and 7.11 g/100g, which was increased by ethanol extraction) and phenolic compounds especially catechin (191.73 μg/mL) which was pronounced in DPP antioxidant potentials (IC_50_ 35.54 mg/g). The FTIR analyses indicated the presence of soluble amides (proteins) and polysaccharides (fibers) functional groups in DPP. Fortification with nanoencapsulated DPP proved to be safe and the recommended form due to the announced positive characteristics. Yoghurt fortification with DPP forms enhanced viscosity, syneresis and Water Holding Capacity (WHC), which can be considered a symbiotic functional product as it contained both probiotics (10^6^ CFU/g) and prebiotics represented in DPP forms.

## Introduction

Yoghurt is one of the most popular dairy products worldwide, which has gained a positive perception as a healthy and natural product, based on health attributes associated with the probiotic effects of yoghurt starter cultures [1]. Yoghurt gels are formed by fermentation of milk using lactic acid bacteria, most commonly used *Streptococcus thermophiles* and *Lactobacillus delbrückii* subsp. *bulgaricus* [2]. Consequently, it has been supplemented with natural food additives to obtain a well-perceived and high-value product [3].

Date palm pollen (DPP) (*Phoenix dactylifera* L.), are the male reproductive cells of palm flowers and commonly used in the Middle East, especially in Egypt. It is considered as an effective natural and functional dietary food supplement due to its remarkable content of bioactive volatile unsaturated fatty acid and flavonoid compounds that play a crucial role as strong antioxidant, anti-breast-cancer, in addition to their nutritional-physiological implications as health-promoting factors that used worldwide as dietary supplements [4,5].

Food products with bioactive compounds, such as polyphenols, have increased in popularity due to their positive role against diseases associated with oxidative stress, such as cancer, cardiovascular and neurodegenerative diseases [6]. Direct incorporation of bioactive compounds into food products is challenging because of their unstable, and low bioavailability, easily oxidable and sensitive to heat and light, which limits their application in the food industry. From this perspective, it was important to preserve their stability, bioactivity and bioavailability [7,8].

Rashidinejad found that the availability of phenolic compounds added to yoghurt decreased due to interactions between phenolic compounds and milk proteins [9]. These interactions were reported by Haratifar and Corredig, who pointed out that these interactions were the main reason for the reduced antioxidant activity of the added phenolic compounds [10].

Nanoencapsulation is widely considered as a useful technology to increase the bioavailability of polyphenols, enhance the stability, lower its toxicity through preventing polyphenols from prematurely interacting with the biological environment, improve intracellular penetration and functionality of bioactive compounds [11,12]. Applying this technology, enabled the reformulation of a wide variety of food products, allowing products’ shelf life elongation and giving new properties supporting their functional bioactive roles [13].

The objective of this study was to evaluate the gross chemical composition, physical and functional potentials of date palm pollen raw grains in comparison with DPP ethanol extract and nanoencapsulated DPP. On the other hand, production of functional yoghurt enriched with DPP three forms to investigate the impact of these fortifications on physicochemical, microstructure, texture and sensory characteristics on the DPP yoghurts, in order to precise the most ideal and functional form of DPP to be consumed to gain its maximum potentials with acceptable sensorial properties.

## Materials and methods

### Chemicals and materials

Fresh cow and buffalo milk was obtained from the Faculty of Agriculture, Alexandria University, Egypt. Reconstituted skim milk (RSM) "DAIRYAMERICA, Inc. California, USA" (34% protein, 51% lactose, 1.2% fat, 8.2% minerals, 4% moisture), was obtained from Alexandria local market. Date palm pollen (DPP) grains was collected ‎from City of Scientific Research and Technological Applications Pilot Farm, Alexandria, Egypt, at the end of spring 2018, and stored in dark glass bottles at ‎‎4°C. Sodium caseinate (NaCas) obtained from American Casein Co. (Burlington, NJ). Soy lecithin and other chemicals were products of Fisher Scientific (Pittsburgh, PA). Folin-Ciocalteu reagent, gallic acid and 1,1-diphenyl—2-pycrylhydrazyl (DPPH) were purchased from Sigma-Aldrich Co. (St. Louis, USA). Lyophilized yoghurt culture YC-X11 consisting of *Lactobacillus delbrueckii* subsp. *bulgaricus* and *Streptococcus thermophilus* (1:1) was obtained from Christen Hansen Laboratories, Copenhagen, Denmark.

### Characterization of DPP grains

#### Gross chemical composition

Total solids, ash, crude fat, crude protein, crude fiber and total sugars were determined for both DPP grains and DPP yoghurt products according to the Official Methods of Analyses [14]. Carbohydrate content was determined as total hydrolysable carbohydrate by the phenol-sulfuric method as described by Dubois *et al* [15]. Concentrations of potassium (K) and minerals; calcium, magnesium, iron, zinc and manganese (Ca, Mg, Zn, Fe and Mn) of DPP grains were determined using Atomic Absorption Spectrometry (AAS) according to [16]. Titratable acidity of DPP fortified yoghurt samples was expressed as an equivalent percentage of lactic acid according to [17].

#### Phenolic content of DPP grains (HPLC)

Separation and quantitative determination of polyphenols content of DPP grains were carried out using HPLC apparatus model 1100 (Agilent Technologies, CA, USA) system column: Agilent Eclipse XDB C18 (150 x4.6 μm 5 μm) according to [18]. The used standards were; gallic Acid, catechin, caffeic acid, rutin, quercetin, cinnamic acid, coumaric acid, ferulic acid, naringenin and propyl gallate.

### Date palm pollen (DPP) extraction and encapsulation

One hundred grams of DPP grains were extracted using ethanol 80% for 24h then homogenized using ‎‎300VT Ultrasonic Homogenizer (BioLogics, Inc. Manassas, VG, USA) at ‎temperature below 25°C [19]. The extracts were filtered and concentrated using rotary evaporator at 45°C. The extract was evaporated under reduced pressure, ‎lyophilized to give a yellow semisolid residue (free extract) (25g) and stored in dark container at 4°C ‎until use.

For encapsulation, Sodium caseinate (NaCas) was hydrated to 5% w/v in deionized water overnight at room temperature (21°C). Soy lecithin 0.5% w/v dissolved in (10 mM sodium phosphate, pH 7). lecithin was mixed with the NaCas solution in ratio 1:1 by gentle magnetic stirring for 1 h. DPP extract was then added to the wall material 10%, and the nanocapsules solution was formed using an Ultra-Turrax homogenizer T18 basic (IKA, Wilmington, USA), operating at a speed of 18,000 rpm for 5 min, ultrasonicated at 160 W power, 20 kHz frequency and with 50% pulse (Sonic Ruptor 400, OMNI International the Homogenizer).

### Nanoencapsulation efficiency (EE) determination

The amount of encapsulated DPP was determined indirectly based on the difference between the total phenolic content of DPP and the free amount of total phenolic content of DPP as described by [20]. To determine the amount of free total phenolic content of DPP, 10 mg of the freeze-dried of DPP-loaded SC-L was mixed with 1 mL of purified water. A vortex was mixed for 30 s and then centrifuged at 11,180 ×g for 5 min. Another 10 mg of the sample of freeze-dried DPP-loaded SC- L was mixed with 1 mL of absolute ethanol to determine the total amount of total phenolic content of DPP. The vortex was mixed for 1 h and then centrifuged at 11,180 ×g for 5 min, and the supernatant was retained. Each extraction was repeated three times, and the supernatants for each type of extraction (e.g. into water or ethanol) were used for total polyphenol measurement.‎ The absorbance of each combined supernatant at 760 nm was determined using ultraviolet-visible spectrophotometry (TU-1810DASPC, Beijing Purkinje General Co., Ltd., Beijing, China). The amounts of total and free phenolic content were determined using a standard curve, which was constructed using standard solutions of 10–100 μg/mL‎ gallic acid in methanol. The total phenolic content was expressed as gallic acid an equivalent (GAE) in mg /g ‎sample.‎The %EE was then calculated as follows:

$$\%EE=\frac{TPC-FPC}{TPC}X\;100$$

Where, EE: Encapsulation efficacy, TPC: Total phenolic content and FPC: Free phenolic content.

### Nanoencapsulated DPP characterization and evaluation

#### Particle size and ζ-potential

The particle size of the nanocapsules was measured using a static light scattering instrument (Mastersizer 2000, Malvern Instruments, Malvern, UK). The particle size of each sample was represented as the surface-weighted mean diameter (d32), which was calculated from the full particle size distribution. The droplet charge (ζ-potential) of the nanocapsules was measured using particle microelectrophoresis (Zetasizer Nano ZS-90, Malvern Instruments, Worcestershire, UK). Samples were diluted with buffer solutions at appropriate pH prior to measurements in order to avoid multiple scattering effects.

#### Microstructural characterization

For preparation of DPP yoghurt samples, cubes (3±0.5mm^3^) were cut from different areas of the yoghurt cup and fixed in 3% glutaraldehyde in 0.05 M phosphate buffer pH 7 for 2 h at 48°C. The fixed cubes were rinsed with 0.05 M phosphate buffer. The fixed cubes were dehydrated by consecutive soaking in 30, 50, 70 and 95% ethanol each for 20 min, and finally was rinsed successively twice by absolute ethanol (100%) at 48°C and 58°C. Cubes were immediately dried in the critical point drier (Samdri PVT-3B, Tousimis, Rockville, MD) for 5h according to Vardhanabhuti [21].

The Microstructure of lyophilized DPP nanocapsules aqueous solutions with different concentrations (20, 30 and 40%) using vacuum freeze-dryer (Model FDF 0350, Korea), as well as the prepared yoghurt samples, were analyzed using a scanning electron microscope (SEM- Joel Jsm 6360LA, Japan) after the surfaces were vacuum coated with gold [22].

#### Fourier transforms infrared (FTIR) spectroscopy

The FTIR spectra of DPP grains, carrier and lyophilized DPP nanocapsules solution (40%) were acquired by using a Fourier transform infrared spectroscopy (Shimadzu FTIR-8400 S, Japan) equipped with (ATR 8000A) in the spectral range of 4000–400 cm^−1^ [23].

#### Fatty acids content via gas-liquid chromatography

The identification of the components of fatty acids methyl esters was performed using gas liquid chromatography using (Hewlett Packard Model 6890 chromatograph), according to Schumann, and Siekmann [24], under the following conditions: Separation was done on an INNO wax (polyethylene glycol) Model No. 19095 N-123, 240°C maximum, capillary column 30.0 m x 530 μm x 1.0 μm, nominal flow 15 mL/ min. with average velocity 89 cm/sec. and pressure 8.2 psi. Column temperature was 240°C with temperature programming: Initial temperature 100°C to 240°C maximum with 10°C rising for each minute and then holds at 240°C/ 10 min. The injection temperature 280°C, back inlet, with split ratio 8:1, split flow120 mL/min., gas saver 20 mL/ min. Carrier gas was nitrogen with flow rate 15 mL/ min. Flame ionization detector temperature 280°C. Hydrogen flow rate 30 mL/ min. Air flow rate 300 mL/min

#### Phenolic, flavonoid content and antioxidant potentials

The total phenolic content was expressed as gallic acid equivalents (GAE) in mg /g sample was determined by the Folin–Ciocalteu method [25].

Total flavonoid content of the aqueous plant extracts was assessed via colorimetric method as described by Sakanaka [26]. The results were expressed as mg of catechol equivalent per g of sample.

The 2,2-diphenyl-1-picrylhydrazyl (DPPH) assay was performed as described by Brand-Williams and co-authors [27]. Antioxidant activity was expressed as an inhibition percent of DPPH radical.

#### Cytotoxicity assessment of nanoencapsulated DPP on RPE1 cell line (BJ1)

Safety of applied nanomaterials represented in carrier (NaCas + Lecithin) and DPP nanocapsules was evaluated against human normal RPE1 fibroblast hTERT-BJ1 cell line via the mitochondrial dependent reduction MTT colorimetric assay according to [28], at the Bioassay-Cell Culture Laboratory, National Research Centre, Egypt.

In a Laminar flow cabinet biosafety class II level (Baker, SG403INT, Sanford, ME, USA), cells were suspended in DMEM-F12 medium, 1% antibiotic-antimycotic mixture (10,000U/mL potassium penicillin, 10,000μg/mL streptomycin sulfate and 25μg/mL amphotericin B) and 1% L-glutamine at 37°C under 5% CO_2_. Cells were batch cultured for 10 days, then seeded at concentration of 10x10^3^ cells/well in fresh complete growth medium in 96-well microtiter plastic plates at 37°C/ 24 h under 5% CO_2_ using a water jacketed carbon dioxide incubator (Sheldon, TC2323, Cornelius, OR, USA). Media was aspirated, fresh medium (without serum) was added and cells were incubated either alone (negative control) or with serial concentration of (100, 50, 25, 12.5, 6.25, 3.125, 1.56 and 0.78 ug/mL) of DPP nanocapsules and carrier (NaCas+ Lecithin). After 48 h of incubation, medium was aspirated, 40uL MTT salt (2.5 μg/mL) were added to each well and incubated for further four hours at 37°C under 5% CO_2_. To stop the reaction and dissolving the formed crystals, 200μL of 10% sodium dodecyl sulphate (SDS) in deionized water was added to each well and incubated overnight at 37°C. DOX were used as positive control at 100μg/mL gives 100% lethality under the same conditions [29]. The cells were examined under light microscope (CKX410Olympus, Japan) at magnification of (X100).

The absorbance was measured using a microplate multi-well reader (Bio-Rad Laboratories Inc., model 3350, Hercules, California, USA) at 595nm and a reference wavelength of 620nm. A statistical significance was tested between the carrier (NaCas+ Lecithin), DPP nanocapsules and negative control using independent t-test by SPSS 11 program. A probit analysis was carried for IC_50_ and IC_90_ determination using SPSS 11 program. The percentage of change in viability was calculated according to the formula:

((S/NC)−1)x100

Where, S: Absorbance of sample and NC: Absorbance of negative control

### Manufacture of DPP yoghurt with different formulations

Cow and buffalo milk was mixed with ratio (1:1) and standardized using skimmed milk powder (SMP) to raise SNF from (8.5) up to (13%). The milk then was homogenized at 200 bar and heat treated at 85°C for 15 min. Hot milk was divided into four equal portions, C; Control plain yoghurt, T_1_; yoghurt enriched with DPP grains, T_2_; yoghurt enriched with DPP ethanol extract and T_3_; yoghurt enriched with DPP nanoencapsulated extract (40%). DPP in all forms was added with enrichment percent of 0.75%, (w/v) of milk. The mix was cooled to 42±1°C then inoculated with (0.03 g/kg) of yoghurt culture YC-X11, poured into 100 mL plastic cups and incubated at 42±1°C until set coagulation at pH ~4.6 (About 5h), then cooled and stored at 4°C [30].

### Physical characteristics of DPP yoghurt

#### Color analyses

Color analyses for DPP yoghurt samples were conducted via Hunter colorimeter (Hunter Ultra Scan VIS). Values were expressed by Hunter *L*, *a*, and *b* values where, L* value of the lightness, as 0–100 representing dark to light, a* value of the degree of red and green color where higher positive indicating more red, and b* value of the degree of the yellow and blue colors, where higher value indicating more yellow [31].

#### The apparent viscosity

The apparent viscosity of the yoghurt was determined by using a Brookfield digital viscometer (Model DV-II + Pro, Brookfield Engineering Laboratories, Middleboro, MA, USA) at 24.8°C with spindle number SC4-15 after 30sec rotation of 80 rpm. Yoghurt samples were stirred for 40 sec before analyzing and results recorded in centipoises (cp) after 50 sec of shearing [32].

#### Syneresis and water holding capacity (WHC)

The yoghurt susceptibility to syneresis and WHC were determined by the method reported by [33]

### Texture profile analyses (TPA)

Textural properties of DPP yoghurt products were evaluated using a texture analyzer (TA1000, Lab Pro (FTC TMS-Pro), USA). Samples were tested in their cups using TA 17 probe (30 mm height and 25 mm diameter), samples were allowed to stand at ambient temperature for at least 1h before testing. A two-bite penetration test was performed and operated at a crosshead speed of 1 mm/sec and penetration distance of 10 mm. Hardness, adhesiveness, cohesiveness, springiness and gumminess were evaluated as described by [34,35].

### Microbiological analyses

The conventional diluting pouring plate technique was used for enumerating microbes in the samples. For total microbial viable count (PCA) of Biolife (Italy) was used, Members of Lactobacilli sp. on MRS agar (Biolife), and the enumeration of yeast and mold was on potato dextrose agar (Biolife) acidified media as described by Standard Methods for the Examination of Dairy Products [36]. The results were calculated directly as colony forming unit (CFU /g).

### Sensory evaluation

Ten panelists, (6 men and 4 women, aged between 27 to 51 years), conducted sensory evaluation on fresh DPP fortified yoghurt samples; plain yoghurt, yoghurt enriched with DPP grains, yoghurt enriched with DPP ethanol extract and yoghurt enriched with DPP nanoencapsulated extract (40%), at Dairy Research Department and Food Technology Research Institute and Food Technology Dept., Arid Lands Cultivation Research Institute, SRTA-City, Alexandria, Egypt as described by [37–39] with some modifications. The criteria for selection depended on their experience and background related to yoghurt products. The samples, which were stored at (4°C), were allowed to rest at room temperature (25°C), 10 min before evaluation. The samples were evaluated using a 10 point Hedonic scale [40]. This scale consisted of the test parameters of flavor, body & texture, appearance & color, odour and overall acceptability, accompanied by a scale of ten categories as: 1 = dislike extremely; 2 = dislike much; 3 = dislike moderately; 4 = dislike slightly, 5 = neither dislike nor like, 6 = like slightly; 7 = like moderately; 8 = like much; 9 & 10 = like extremely.

### Statistical analyses

All data were expressed as mean values ± SD. Statistical analyses were performed via Statistical Analyses System (SAS) software program (SAS Institute 2004). Statistical analyses were performed using one-way analyses of variance (ANOVA) followed by Duncan’s test. Sensory properties were analyzed statistically by two-way analyses of variance using (ANOVA) followed by t-test (LSD). Differences were considered significant at *p* < 0.05.

## Results and discussion

### Characterization of DPP grains

#### The gross chemical composition

Table (1) illustrates the gross chemical composition of Egyptian DPP grains. The calculated energy value was 310.88 Kcal/ 100g. Results indicated that protein, carbohydrate and fat represented 40%, 19% and 12%, respectively of DPP total solids content. Nutrient results were in agreement with Hassan [41].

**Table 1:** Gross composition of DPP grains.

Component	Content	%DV
**Energy**	310.88 **Kcal.**	
**Nutrients**	**g/100g**	
Total solids	91.11±0.43	--
Ash	10. 23±0.02	--
Crude fat	10.80±0.03	16.61
Crude protein	36.28±0.57	72.56
Crude fiber	8.09±0.13	32.36
Total sugar	6.50±0.69	13.00
Carbohydrate	17.14±0.47	5.71
**Minerals**	**mg/100g**	
Calcium (Ca)	560.00	56.00
Potassium (K)	750.00	21.42
Magnesium (Mg)	318.70	79.67
Iron (Fe)	226.50	1258.33
Zinc (Zn)	124.40	829.33
Manganese (Mn)	70.00	17.50

Nutrients data represented the means ± standard deviation, n = 3

Table (1), also shows that DPP constitute a rich source of mineral elements. The main mineral on concentration basis was potassium 750 mg/100g, followed by calcium 560 mg/100g, magnesium 318.7 mg/100g, and iron 226.5 mg100/g. DPP also contain useful amount of zinc 124.4 mg/100g, manganese and 70 mg/100g. On the other hand, Comparing according to daily value basis revealed that 100g of examined DPP provide an excessive amount of iron and zinc (1258.33 and 829.33%DV, respectively) and more than half daily requirements of magnesium and calcium (79.6756 and 56%DV). Being a good source of minerals such as zinc, iron made the date palm pollen to be related with stimulation of sperm motility and the progressive forward movement [42].

#### Identification of phenolic compounds (HPLC)

The findings (Table 2) revealed the presence of ten polyphenols compounds, namely gallic acid, catechin, caffeic acid, rutin, quercetin, cinnamic acid, coumaric acid, ferulic acid, naringenin and propyl gallate. Those compounds were partially identified by the comparison of their retention times to those of authentic standards analyzed under identical conditions. The results indicate that DPP contained 19.20 μg/ml gallic acid, 191.73 μg/ ml catechin, 1.74 μg/ ml coffeic acid, 3.71 μg/ ml rutin, 3.91 μg/ ml quercetin, 0.46 μg/ ml cinnamic acid, 0.56 μg/ ml coumaric acid, 0.57 μg/ ml ferulic acid, 0.54 μg/ ml naringenin and 0.51 μg/ ml propyl gallate, similar to Abed El-Azim [43]. While Daoud reported reach phenolic content of various extracts of two Tunisian cultivars DPP [44]. Catechins are flavonoid compounds found in a variety of plant. Catechins showed to be the major phenolic compounds in DPP. These results were in agreement with what previously reported by Grzesik [45].

**Table 2:** Phenolic compounds profile of DPP extract.

Phenolic compound	Concentration (μg/ml)
Gallic Acid	19.20
Catechin	191.73
Caffeic Acid	1.74
Rutin	3.71
Quercetin	3.91
Cinnamic Acid	0.46
Coumaric Acid	0.56
Ferulic Acid	0.57
Naringenin	0.54
Propyl Gallate	0.51

Gas-Liquid Chromatographic analysis of fatty acids content

Gas-Liquid Chromatographic analysis of fatty acids contents of DPP before and after 80% ethanol extraction are represented in Table 3. Results showed that the lipids fraction in the DPP grains and DPP ethanol extract included 11 fatty acids. The DPP grains content of SFA was: palmitic, myristic, arachidic, lauric, stearic and capric, arranged in a descending order according to concentrations 24.24, 16.22, 6.64, 5.08, 3.43 and 0.46 g/ 100g, respectively. Ethanol extraction of DPP grains was found to suppress the SFA content especially myristic, arachidic and lauric to 0.75, 1.09 and 0.85 g/100g, respectively. These results agreed with Lima [46]. Palmitoleic and oleic acids represented the monounsaturated fatty acids in the DPP that exert the good flavor as previously reported [47]. Unsaturated fatty acids constitute of both DPP grains and extract represented 43.93 and 68.39%, respectively of total fatty acids content, with dominance of oleic acid (ω-9) (7.11 and 12.15%) and linoleic acid (ω-6) (20.26 and 35.38%), respectively. Noteworthy that extraction of DPP increased the levels of unsaturated fatty acids content (especially ω-9, ω-6 and ω-3) and decreased the saturated fatty acids content by almost two folds as pronounced in UFAs: SFAs ratio of DPP grains and extract that recorded (0.78: 1 and 2.16:1, respectively). Furthermore, extraction raised the ω6/ ω3 ratio from 2.31 up to 2.83. Optimal dose or ratio of omega-6/omega-3 is an important determinant of health that varies from 1/1 to 4/1, as appropriate amounts of dietary omega-6 and omega-3 fatty acids are needed to be considered in making dietary recommendations, and should be distinguished in food labels because they are metabolically and functionally distinct [48,49].

**Table 3:** Gas-Liquid chromatographic analysis of fatty acids content.

Components	Symbol	DPP grains (g/100g)	DPP extract (g/100g)
**SFAs**			
Capric acid	(Cl0:0)	0.46	0.84
Lauric acid	(Cl2:0)	5.08	0.85
Myristic acid	(Cl4:0)	16.22	0.75
Palmitic acid	(Cl6:0)	24.24	24.89
Stearic acid	(Cl8:0)	3.43	3.19
Arachidic acid	(C20:0)	6.64	1.09
**USFAs**			
**MUFAs**			
Palmitoleic acid	(Cl6:l n-7)	7.23	7.5
Oleic acid	(Cl8:l n-9)	7.11	12.15
**PUFAs**			
Linoleic acid	(Cl8:2 n-6)	20.26	35.38
Linolenic acid	(Cl8:3 n-3)	8.76	12.52
Arachidonic acid	(C20:4 n-6)	0. 57	0.78
**SFAs**		56.07	31.61
**UFAs**		43.93	68.39
**MUFAs**		14.34	19.71
**PUFAs**		29.59	48.68
**PUFAs: MUFAs ratio**		2.06	2.47
**UFAs: SFAs ratio**		0.78: 1	2.16:1
**ω6/ ω3 ratio**		2.31	2.83

SFAs; Saturated fatty acids, USFAs; Unsaturated fatty acids, MUFAs; Monounsaturated fatty acids, PUFAs; Polyunsaturated fatty acids

### Nanoencapsulation efficiency

Table (4) illustrated the encapsulation efficiency of the DPP extractions with ratios of 20, 30, and 40 mg/ g NaCas-L. The encapsulation efficiency was 93.78%, 91.70%, and 89.65% for ratios of 20, 30, and 40 mg/ g NaCas-L, respectively. It was observed that there was no significant difference between the concentration 20 and 30 mg in loading efficiency which indicated that 5% w/v NaCas reached its encapsulation capacity under the conditions studied. The encapsulation efficiency as determined decreased to around 89.65% with 40 mg/g NaCas and did not decrease significantly because lecithin may be working to increase the efficiency of loading, especially for the hydrophobic phase. These results are in agreement with Pan and Rezaei [50,51]. The NaCas-HMP and NaCas-CMC showed high encapsulation efficiency for curcumin and a better choice for a product where transparency is needed [52].

**Table 4:** Size (nm) and ζ potential (mV), ‎polydispersity index (PDI), and encapsulation efficiency‎ for NaCas-L and DPP encapsulated.

Sample	Size (nm)	Calculated PDI	ζ potential (mV)	Encapsulation Efficiency %
0 DPP	174.60	0.220	-22.3	--
20 DPP	198.30	0.468	-10.50	93.78± 3.24^a^
30 DPP	249.60	0.527	-10.60	91.70 ± 2.31^a^
40 DPP	274.90	0.531	-10.70	89.65 ± 3.15^b^

Results are means ± standard deviation for triplicates.

Different superscripts indicate differences in the means (*p* < 0.05).

NaCas-L dispersions at pH 7.0 with DPP extraction had a much lower magnitude of ζ potential when compared with NaCas alone, and the DPP concentration did not affect the ζ potential significantly (Table 4). Theoretically, casein particles are expected ζ potential more negatives because of a smaller quantity of κ-casein per particle. The ζ potential results in the Table (4) agree with this expectation based on the particle size. The ζ potential high magnitude can prevent particle aggregation during storage. The sizes of NaCas-L and the encapsulated DPP followed a normal distribution curve but with the broader right side in case of encapsulated DPP indicating the presence of ‎large size particles. This was also apparent from comparing the mean particle size and the ‎PDI of the NaCas-L and the encapsulated DPP (Table 4). Increasing the percentage of loaded DPP ‎increased the size of the capsules (p<0.05). However, the size of 20 mg entrapped DPP ‎particles was comparable to that of NaCas-L.

### Characterization of nanoencapsulated DPP

#### Microstructural characterization

**Fig 1:**
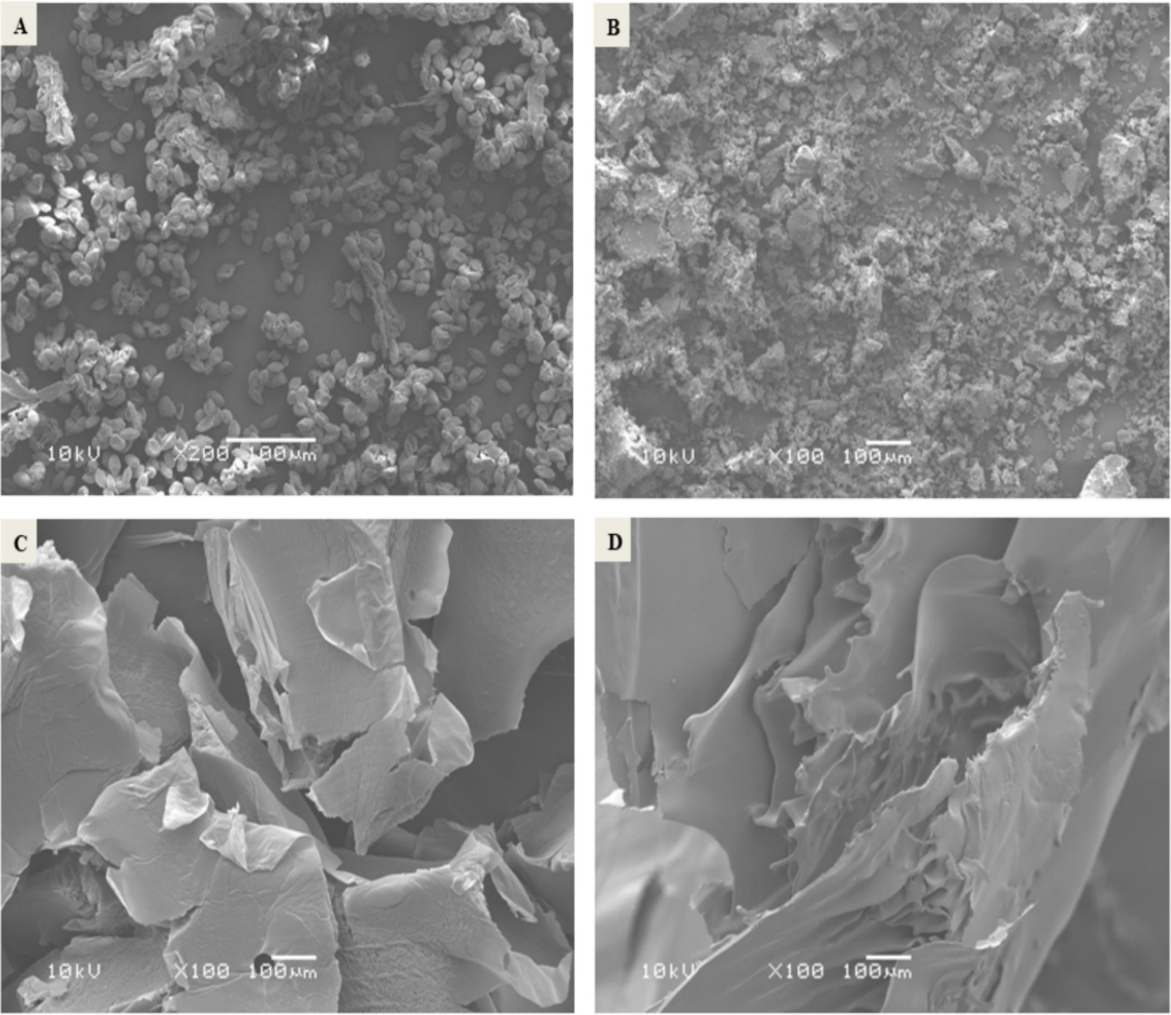
SEM micrographs of date palm pollen grains and nanoencapsulated DPP with different extract concentrations. Date palm pollen grains (100μm, X200, 10kV) (A). Nanoencapsulated DPP with extract concentration 20% (100μm, X100, 10kV) (B). Nanoencapsulated DPP with extract concentration 30% (100μm, X100, 10kV) (C). Nanoencapsulated DPP with extract concentration 40% (100μm, X100, 10kV) (D).

Fig 1A, 1B, 1C and 1D showed SEM micrograph of Egyptian date palm pollen grains (100μm, X200, 10kV) and freeze-dried nanoencapsulated DPP with different extract concentrations 20%, 30% 40% at the same magnification (100μm, X100, 10kV). In the micrograph (Fig 1A), the Egyptian DPP grains appeared relatively uniform, smooth surface and oval-shaped with a longitudinal groove which may play an important role as a diagnostic mark of the plant. The grain dimensions were 19± 0.02μm and 7± 0.07μm for long and short axes, respectively. Similar observations were reported concerning DPP grains originated from United Arab Emirates (UAE) and Iran [53,54].

Micrographs Fig 1B, 1C and 1D indicated that microstructure of nanoencapsulated DPP was affected by increasing extract concentration to become softer and more intact with fewer gaps between its particles. On the other hand, freeze-drying of natural biopolymers originated from plants was reported to exert better microstructure that leads to better performance of functional properties [37].

#### Fourier transforms infrared (FTIR) spectroscopy

**Fig 2:**
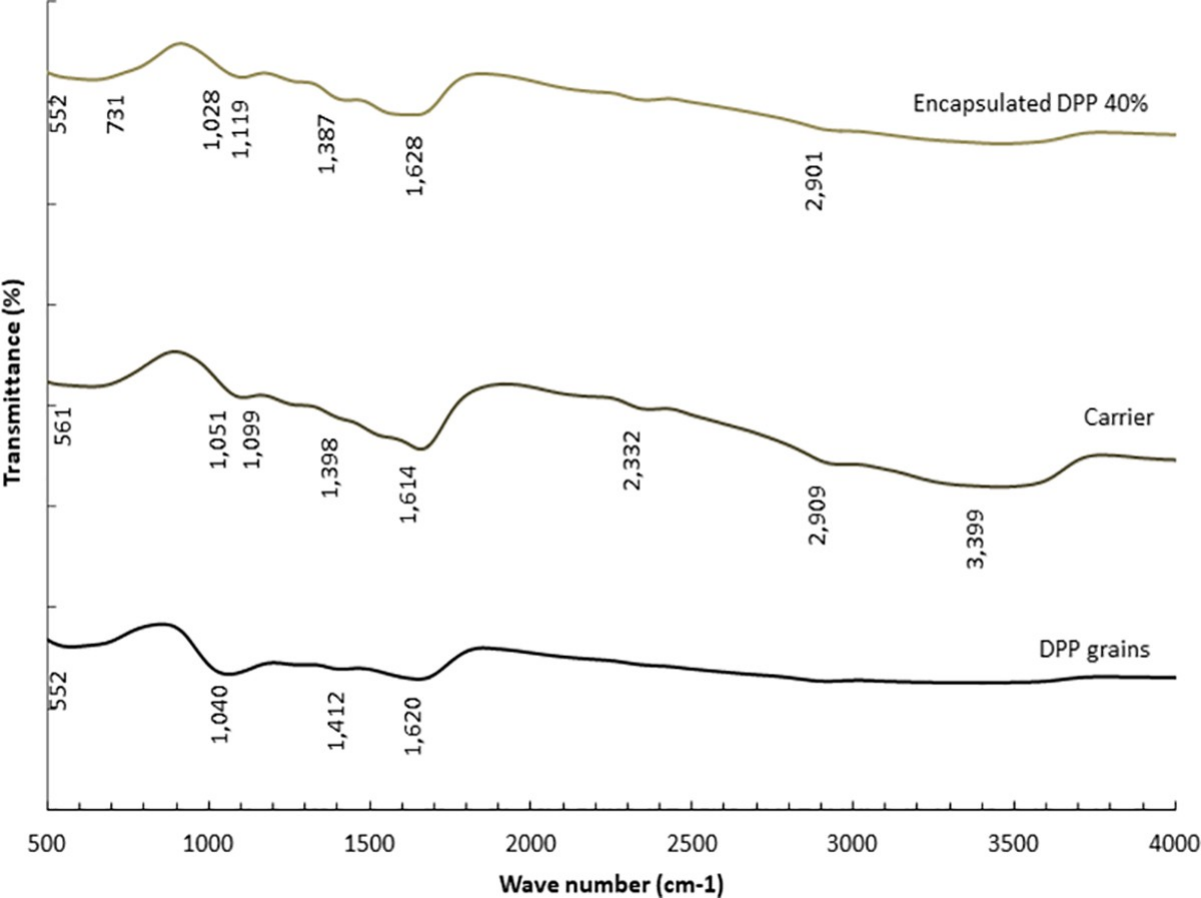
Fourier transform infrared spectrophotometer (FTIR) of DPP grains, carrier and encapsulated DPP (40%).

Fig (2) shows IR spectra graphs of DPP grains, carrier and encapsulated DPP (40%) in order to facilitate marking changes in functional groups with extraction and encapsulation. The IR spectra articulated that DPP functional groups did not affect with extraction and encapsulation procedures. Furthermore, the most displayed functional groups in IR spectra of DPP either in grains or encapsulated forms, were in the region between 1630 and 1000 cm^-1^ that represent soluble amides (proteins) and polysaccharides (fibers) functional groups. Some plants was reported to be a complex polymeric substances of carbohydrate nature with branched structure of polar glycoprotein and exopolysaccharides [37,55]. These results are in convenience with gross chemical composition represented in (Table 1), where protein and carbohydrates represent the main constituents of DPP grains.

#### Total phenolic, total flavonoids content and antioxidant potentials

Table (5) represent phenolic, flavonoid content and antioxidant potentials of DPP grains, ethanol extract and encapsulated DPP in addition to the impact of their fortification in yoghurt products. DPP forms can be arranged in descending order according to total phenolic content as follows, DPP ethanol extract, encapsulated and DPP grains (74.9, 43.19 and 17.43 mg/ g). This may be attributed to the initial high phenolic content of DPP grains (Table 2) which increased by ethanol extraction. Same pattern was announced in flavonoid content. Antioxidant scavenging potentials are represented with IC_50_ (mg/ ml), the inhibitory concentration at which 50% of DPPH radicals are scavenged. Results revealed that the highest antioxidant potential amongst the three forms of DPP was the 80% ethanol extract followed by encapsulated form then The DPP grains with values of (11.76, 19.56 and 35.54 mg/ ml, respectively). DPP antioxidant potentials was previously documented [44,56]. Fortifying with these forms of DPP reflected the same pattern on the yoghurt products exerting functional properties.

**Table 5:** Phenolic content and antioxidant potentials.

Sample	Total phenolic*	Total flavonoids*	Antioxidant (DPPH) (IC_50_) **
DPP grains	17.43±0.16	13.16±0.20	35.54±0.43
DPP ethanol extract	74.90±0.55	26.28±0.81	11.76±0.35
DPP encapsulated	43.19±0.75	13.78±0.47	19.56±0.16
C (Control)	5.47±0.36	12.08±0.01	584.33±0.74
T_1_ (DPP grains)	8.85±0.59	12.26±0.09	216.38±0.81
T_2_ (DPP ethanol extract)	27.50±0.14	17.46±0.18	45.09±0.48
T_3_ (Nanoencapsulated DPP)	14.27±0.74	12.26±0.09	141.77±0.91

Results represent means of duplicates ±SD

*Total phenolic total flavonoids contents are expressed as mg/ g sample

**IC_50_ (mg/g): Inhibitory concentration at which 50% of DPPH radical is scavenged

C: Control plain yoghurt, T_1_; Yoghurt enriched with DPP grains, T_2_; Yoghurt enriched with DPP ethanol extract and T_3_; Yoghurt enriched with DPP nanoencapsulated extract (40%)

#### Cytotoxicity assessment of nanoencapsulated DPP on RPE1 cell line (BJ1)

**Fig 3:**
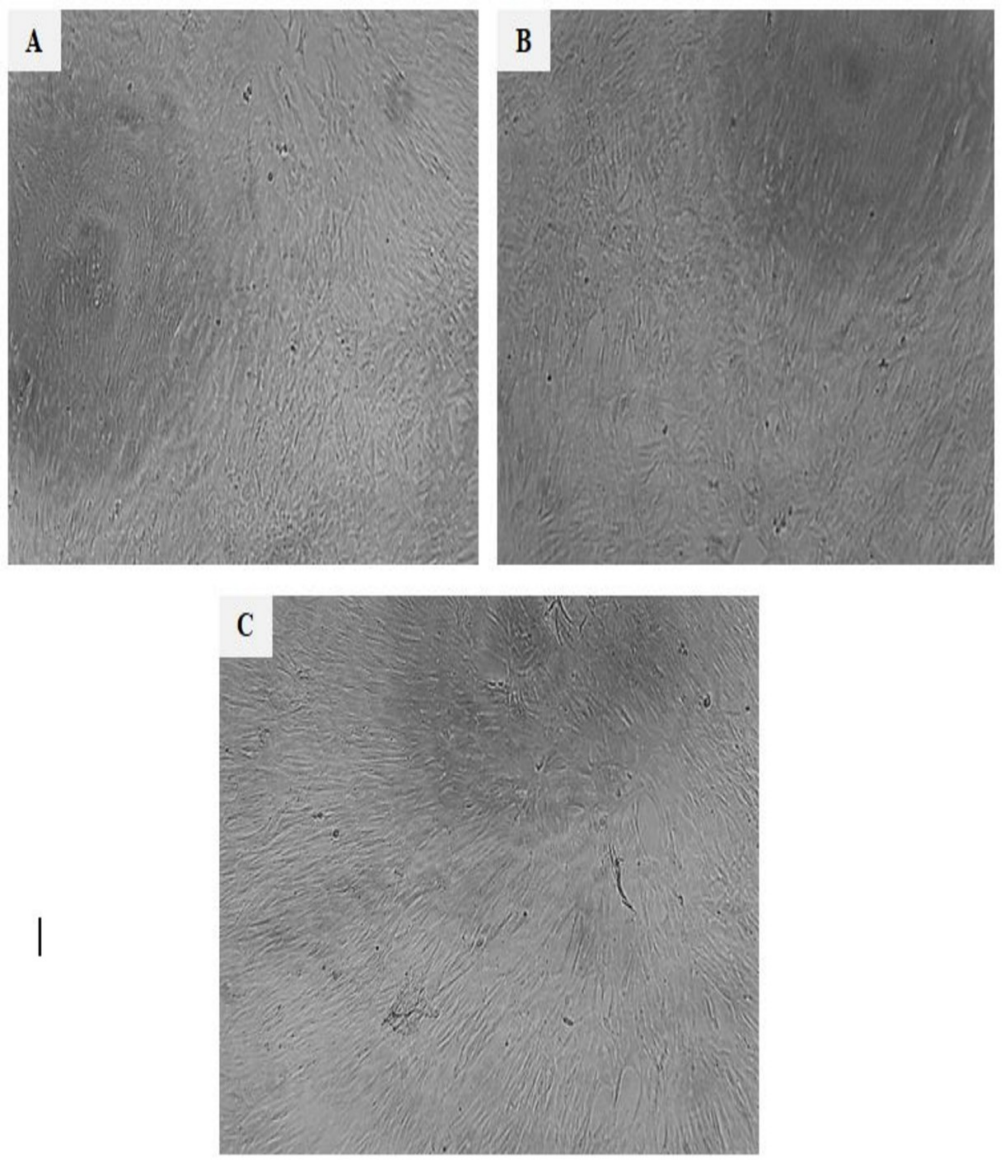
Morphological examination of RPE1 normal human retina cells. Light microscopy observation of treated RPE1 normal human retina cells using concentrations of 100 μg/ mL after 48h (X100). RPE1 negative control (A). RPE1 treated with carrier (NaCas + Lecithin) (B). RPE1 treated with DPP nanocapsules (C).

Table (6) illustrated the cytotoxicity of carrier (NaCas+ Lecithin) and DPP nanocapsules against human normal RPE1 fibroblast hTERT-BJ1 cell line. The results revealed that the used materials did not show any adverse effects on RPE1 cells up to 100 μg/mL with incubation for 48h, and percentage of cell death did not exceed 1.2% and 10.3% at the higher applied concentration 100 μg/mL in both carrier (NaCas+ Lecithin) and DPP nanocapsules treated cells respectively. Accordingly, morphological examination of RPE1 normal human retina cells on light microscope shown in Fig 3A, 3B and 3C, did not show any morphological changes in treated cells using concentration of 100 μg/mL and incubation for 48h, which appeared similar in architecture to negative control. The obtained results agreed with [57,58], who reported the safety of lecithin and date palm pollen. These results revealed the safe use of carrier and DPP nanocapsules which encourage their food applications.

**Table 6:** Cytotoxicity assessment of DPP nanocapsules on human normal RPE1 (BJ1) cells.

Sample	IC_50__*_	IC_90__**_	Percentage of cell death
Negative control	ND	ND	0%
Carrier (NaCas+ Lecithin)	ND	ND	1.2% at 100 μg/mL
DPP nanocapsules	ND	ND	10.3% at 100 μg/mL

*IC_50_ μg/mL): Lethal concentration which causes the death of 50% of cells in 48 h

**IC_90_ (μg/mL): Lethal concentration which causes the death of 90% of cells in 48 h

Sample concentrations range (100 to 0.78 μg/mL)

ND: Not detected

### Physicochemical properties of DPP yoghurt types

#### Chemical characterization

Gross chemical composition of yoghurt types fortified with different DPP forms including total solids, fat, protein, total sugars, ash and acidity are illustrated in Table 7. Results revealed insignificant increase in total solid and acidity in the enriched DPP yoghurt (T_1_, T_2_ and T_3_) compared to control. DPP enrichment did not noticeably affect other chemical parameters, fat, protein, total sugars and ash contents, due to small fortification percent (0.75% w/v) of milk. These results are in accordance with previously reported by Metry and Yerlikaya [59,60].

**Table 7:** Physicochemical characteristics of DPP yoghurt.

Parameter	Control	T_1_	T_2_	T_3_
Chemical characterization (g/100g)				
Total solids	15.19±0.08^a^	16.16±0.15^a^	16.56±1.09^a^	16.77±0.27^a^
Fat	3.30±0.07^a^	3.50±0.03^a^	3.40±0.03^a^	3.30±0.00^a^
Protein	3.21±0.04^a^	3.30±0.03^a^	3.27±0.21^a^	3.17±0.14^a^
Total sugars	4.76±0.03^b^	4.54±0.03^b^	4.78±0.01^b^	4.50±0.14^b^
Ash	1.09±0.02^b^	1.11±0.12^b^	1.13±0.03^b^	1.18±0.37^b^
Titratable Acidity	0.87±0.01^b^	0.92±0.02^b^	0.90±0.02^b^	0.95±0.01^b^
Physical characterization (Colour analyses)				
L*	88.9±0.81^a^	83.14±0.46^b^	87.92±0.62^a^	88.28±0.43^a^
a*	1.85±0.30 ^a^	0.11±0.03^b^	2.07±0.42^a^	2.33±0.18^a^
b*	8.33±0.64 ^a^	10.31±0.33^b^	10.38±0.26^b^	8.46±0.73^a^

Data presented are the means of duplicates ±SD

^a,b,..^Mean in the same row followed by different superscript letters differ significantly (*p*<0.05)

L*, value measuring black (0)/white (100); a*, value measuring green (-)/red (+); b*, value measuring blue (-)/yellow (+)

Control plain yoghurt, T_1_; Yoghurt enriched with DPP grains, T_2_; Yoghurt enriched with DPP ethanol extract and T_3_; Yoghurt enriched with DPP nanoencapsulated extract (40%)

#### Physical characterization

The color analyses of fortified DPP yoghurt are illustrated in Table 7. T_1_ showed significant decrease in both Lightness (L) and a with increased b values compared to control. This result indicates darker yellowish color that matched the sensory evaluation. T_2_ also showed to be yellowish as it affected b values to significant increase. The color differences in T_1_ and T_2_ in DPP grains and ethanol extract forms used to enrich yoghurts mainly can be relied to brownish yellow color of used DPP. On the other hand, fortifying with encapsulated DPP form did not indicate any differences in color with control. These results are supported with sensory evaluation results.

**Fig 4:**
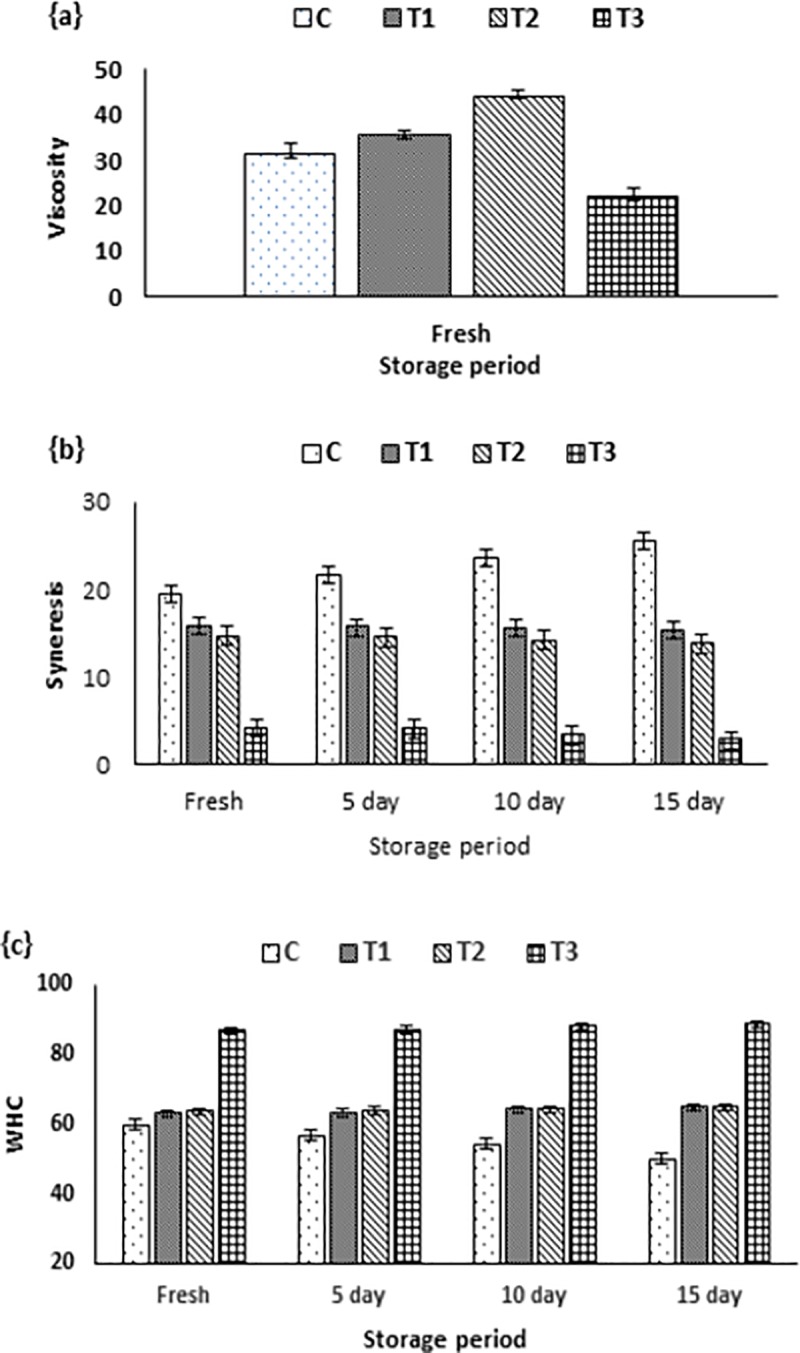
Physical characteristics of fortified yoghurt. Viscosity (a). Syneresis (b). water-holding capacity (WHC) (c). C: Control plain yoghurt, T_1_; Yoghurt enriched with DPP grains, T_2_; Yoghurt enriched with DPP ethanol extract and T_3_; Yoghurt enriched with DPP nanoencapsulated extract (40%).

Fig (4A) illustrated viscosity in fortified DPP yoghurt. The viscosity values tended to increase in T_1_ and T_2_ (fortified with DPP grains and ethanol extract, respectively), while viscosity values decreased in T_3._ This phenomenon may be explained as the NaCas and Lecithin in the encapsulated DPP form enhanced the phase separation and consequently lowered the viscosity [61,62].

Fortifying yoghurt with different DPP forms decreased syneresis and increase WHC especially in the encapsulated form (T_3_) as shown in Fig 4B and 4C. The relation trend of expelled whey (syneresis) and WHC in all samples showed to be inversely proportional. The elevation of total solids of DPP enriched yoghurt (Table 7) increased the bound water (WHC) which consequently suppressed water separation. Low WHC and whey separation in plain control yoghurt may be related to unstable gel network with extreme rearrangements [63].

### Texture profile analyses (TPA) of DPP yoghurt types

**Fig 5:**
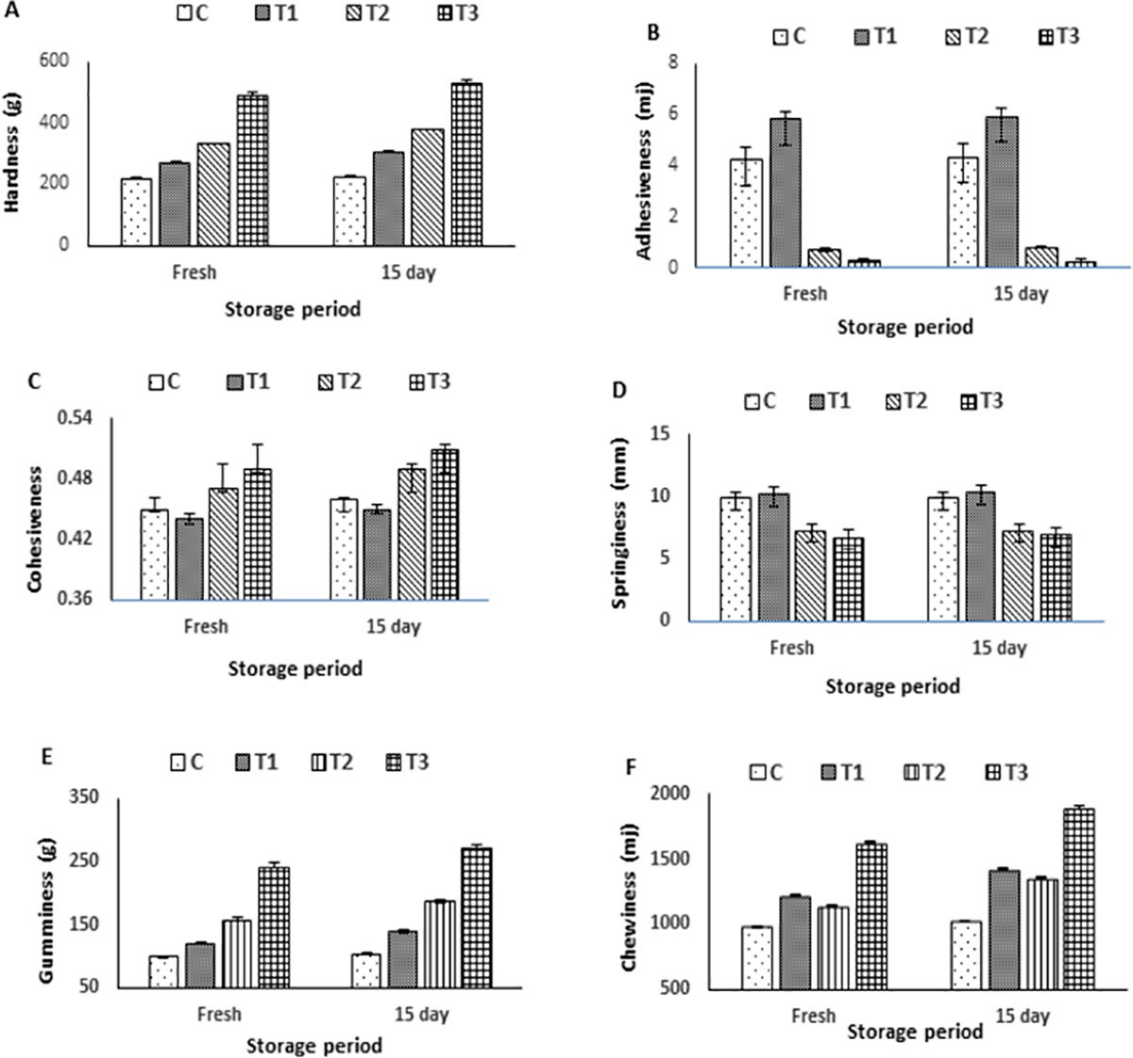
Texture profile analyses of DPP yoghurt with different forms during the storage. Hardness (A). Adhesiveness (B). Cohesiveness (C). Springiness (D). Gumminess (E). Chewiness (F). C: Control plain yoghurt, T_1_; Yoghurt enriched with DPP grains, T_2_; Yoghurt enriched with DPP ethanol extract and T_3_; Yoghurt enriched with DPP nanoencapsulated extract (40%).

Texture profile analyses of DPP yoghurt with different forms during the storage are exhibited in (Fig 5A–5F representing hardness, adhesiveness, cohesiveness, springiness, gumminess and chewiness, respectively. Fortifying with of DPP forms to be involved in the yoghurt matrix, affected positively the TPA of enriched yoghurt. T_1_, T_2_ and T_3_ DPP fortified yoghurt types exhibited differences in their behavior as compared to control yoghurt (C). Either fresh or along the storage period, enriched yoghurt recorded higher values of the hardness, cohesiveness, gumminess and chewiness as compared to control yoghurt. On the contrary, adhesiveness and springiness of fortified yoghurt types decreased in comparison with control yoghurt. The increase of hardness and cohesiveness could be due to the reduction of pH during storage, causing the gel to contract and consequently increased gel firmness [64]. The results revealed an inverse relationship between adhesiveness and hardness. In addition, the increased hardness may result in improvement of the yoghurt texture making it less susceptible to rearrangements within its network and consequently less susceptible to shrinkage and serum expulsion, which were previously reported [59,65]. According to the texture analyses data, yoghurt fortified with the nanoencapsulation DPP form (T_3)_ exhibited the best texture among other fortified types and control.

### Microstructural characterization

**Fig 6:**
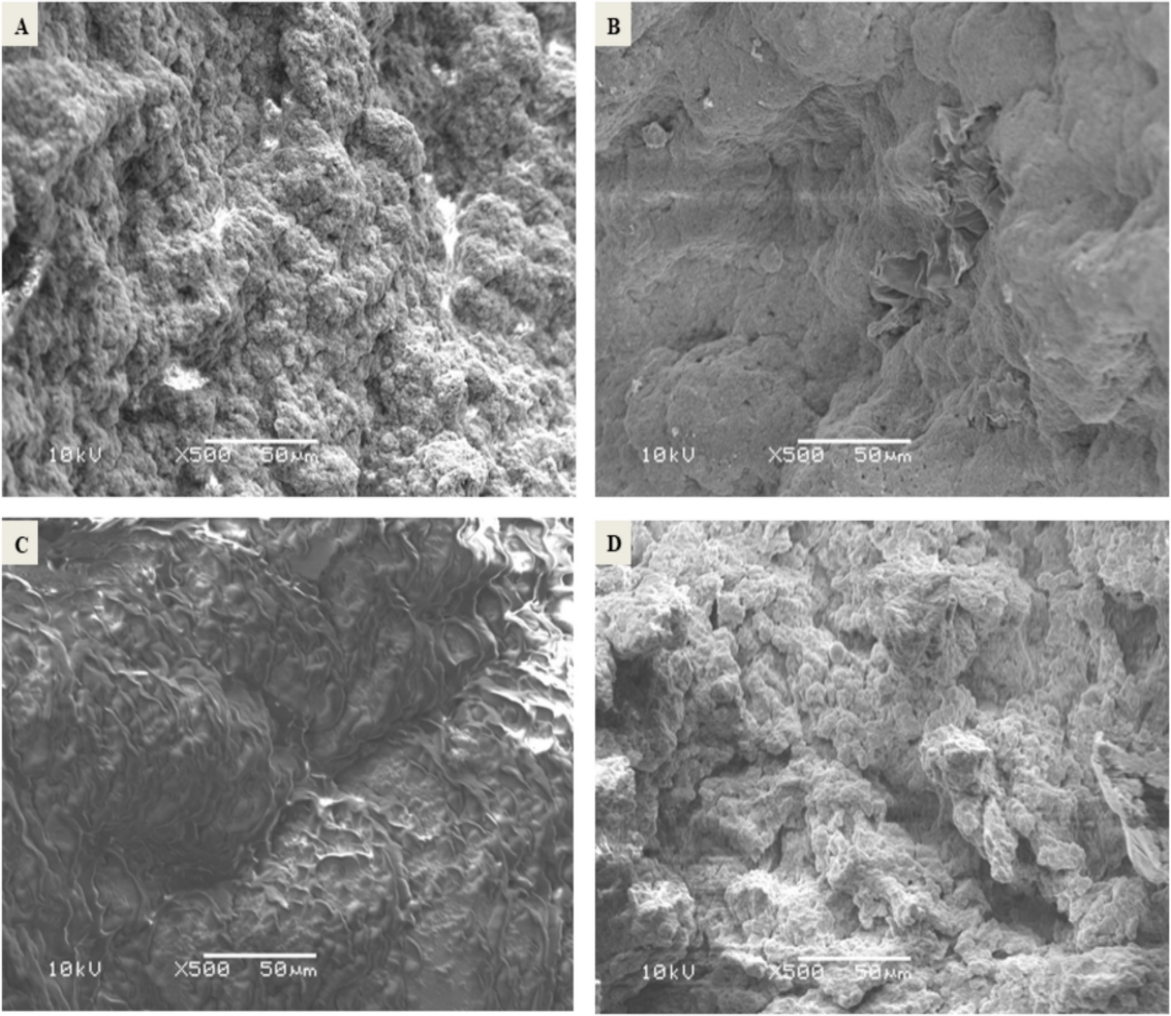
SEM micrographs DPP fortified yoghurt with different forms (50μm, X500, 10kV). Control plain yoghurt (A). Yoghurt enriched with DPP grains T_1_ (B). Yoghurt enriched with DPP ethanol extract T_2_ (C). Yoghurt enriched with DPP nanoencapsulated extract (40%) T_3_ (D).

Fig 6A, 6B, 6C and 6D illustrated DPP fortified yoghurt with different forms control plain yoghurt, yoghurt enriched with DPP grains T_1_, yoghurt enriched with DPP ethanol extract T_2_ and yoghurt enriched with DPP nanoencapsulated extract (40%) T_3,_ respectively (50μm, X500, 10kV). Nanoencapsulated DPP fortified yoghurt (Fig 6D) showed to be more similar to control (Fig 6A) while other fortification forms (Fig 6B and 6C) revealed intact sheets like structure which could significantly affect sensory evaluation. This result indicated that nanoencapsulated DPP was able to arrange through yoghurt structure with less effect.

### Microbiological analyses

Microbiological analyses of DPP fortified yoghurt with different forms including total viable counts, *Lactobacilli* sp. counts and yeast and molds (CFU/ g) are illustrated in (Table 8). The main aim of microbiological analyses of fortified yoghurt types was to ensure that these fortifications do not represent obstructions in the viability of the lactic acid bacteria and yoghurt starter culture represented in *Lactobacilli* sp. group, in addition to the assessment to the best-before date. This target was achieved as obtained results of the three forms of DPP fortification did not significantly affect viable counts of either total or *Lactobacilli* group counts comparing to control unfortified yoghurt type along the storage period. Additionally, yeast and molds were not detected in all treatments along the 15 storage days which indicate good hygienic conditions of processing. Noteworthy, that total viable counts of fortified yoghurt types exceeded 10^6^ CFU/ g (till the tenth day of storage which nominate these yoghurts to be symbiotic functional products containing the recommended count of viable probiotics (10^6^ CFU/ g) and fortified with DPP prebiotic. As a guide, the International Dairy Federation (IDF) suggested a minimum of 10^6^ CFU of probiotics/g product should be alive at the time of consumption [66]. Upon the obtained results the DPP fortified yoghurt products are recommended to be used best-before 10 days of production date.

**Table 8:** Microbiological analyses of DPP fortified yoghurt with different forms.

Treatments	Zero time	5 days	10 days	15 days
Total viable count (CFU g^-1^)				
Control	1.7 X10^6^	1.9 X10^6^	2.1 X10^6^	1.5 X10^5^
T_1_	1.6 X10^6^	1.8 X10^6^	2.2 X10^6^	1.7 X10^5^
T_2_	1.7 X10^6^	1.8 X10^6^	1.9 X10^6^	6.2 X10^4^
T_3_	1.8.X10^6^	2.3 X10^6^	2.6 X10^6^	2.2 X10^5^
*Lactobacilli* sp. (CFU g^-1^)				
Control	5.3 X10^3^	5.5 X10^3^	5.7 X10^3^	3.7 X10^3^
T_1_	5.4 X10^3^	5.8 X10^3^	6.3X10^3^	4.5 X10^3^
T_2_	5.2 X10^3^	5.4 X10^3^	5.5 X10^3^	8.7 X10^2^
T_3_	5.1 X10^3^	5.9 X10^3^	6.4 X10^3^	4.7 X10^3^
Yeast & Molds (CFU g^-1^)				
Control	ND	ND	ND	ND
T_1_	ND	ND	ND	ND
T_2_	ND	ND	ND	ND
T_3_	ND	ND	ND	ND

Control plain yoghurt, T_1_; Yoghurt enriched with DPP grains, T_2_; Yoghurt enriched with DPP ethanol extract and T_3_; Yoghurt enriched with DPP nanoencapsulated extract (40%)

### Sensory evaluation

**Fig 7:**
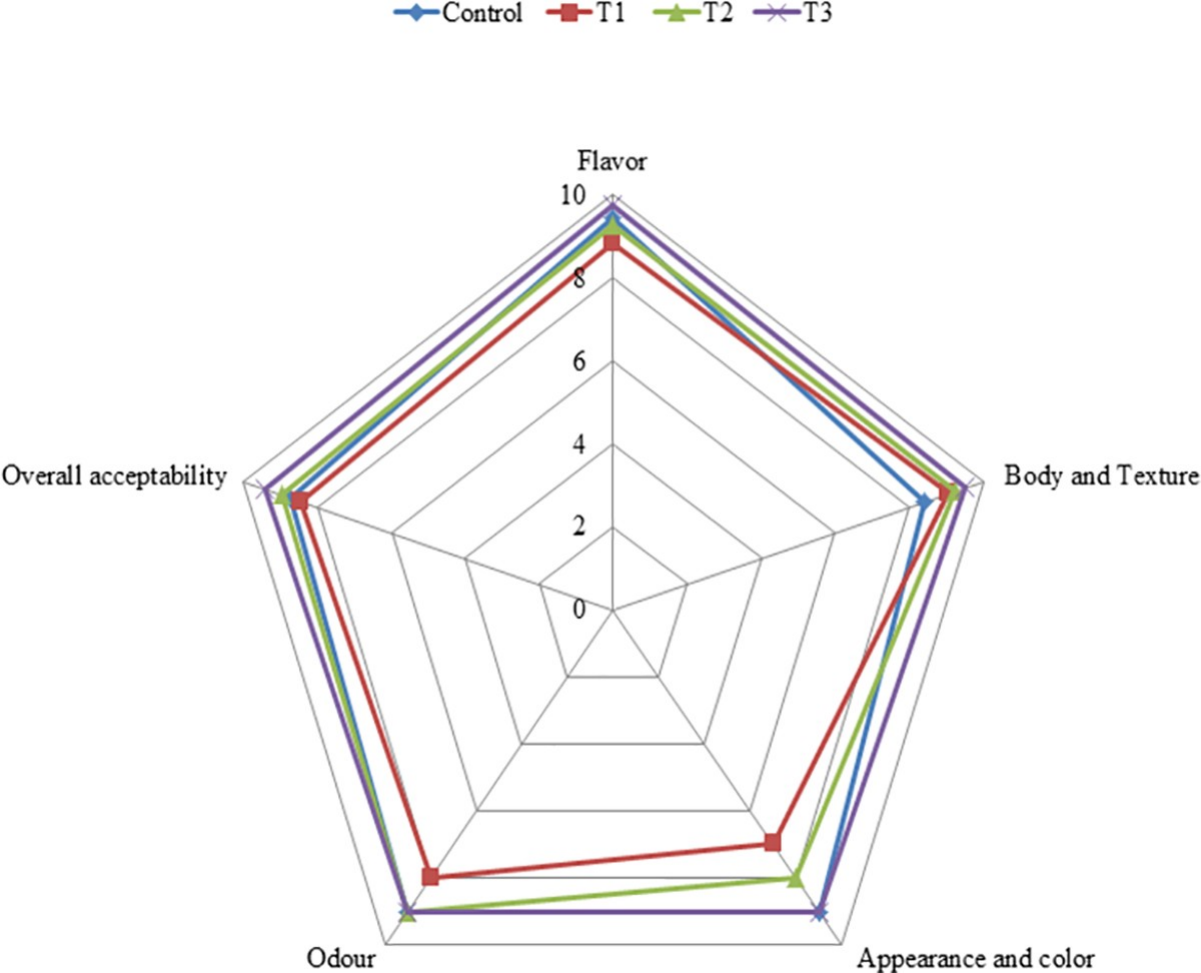
Sensory evaluation of fresh DPP fortified yoghurt with different forms. Control plain yoghurt, T_1_; Yoghurt enriched with DPP grains, T_2_; Yoghurt enriched with DPP ethanol extract and T_3_; Yoghurt enriched with DPP nanoencapsulated extract (40%).

The sensory evaluations of fresh DPP fortified yoghurt with different forms are illustrated in (Fig 7). Generally, there were significant differences (p<0.05) among treatments for flavor, body & texture and overall acceptability of sensory evaluation during storage. Yoghurt enriched with DPP grains (T_1_) scored the lowest total scores. This could be related to yellowness showed by T_1_ and T_2_ enriched yoghurt fortified with DPP grains and ethanol extract, respectively which was correlated with higher b values of their yoghurt types (Table 7) and impact on microstructure illustrated in (Fig 2). On contrary, T_3_ scored the best scores of appearance and body and texture which can be connected with microstructure results (Fig 2). However, fresh yoghurt enriched with encapsulated DPP (T_3_) showed to be sensorial preferred as its overall acceptability value (9.43) was significantly higher than T_1_ (8.45) and T_2_ (8.95), and to control (8.73). Sensory evaluation of DPP yoghurt with different forms during storage represented in (Table 9), reflected that significant decrease in overall acceptability could be relied mainly to flavor that was the only sensory feature significantly affected along the 15 days of storage. Based on obtained sensory data, nanoencapsulated DPP could be recommended for fortified yoghurt production with high quality sensorial aspects.

**Table 9:** Sensory evaluation of DPP yoghurt with different forms during storage.

Treatments	5 days	10 days	15 days	Mean
**Flavor**				
Control	9.8 ± 1.49	9.3 ± 1.33	8.9 ± 1.81	**9.37** ^**B**^
T_1_	9.1 ± 1.49	8.7 ± 1.76	8.1 ± 1.49	**8.75**^**D**^
T_2_	9.5 ± 1.33	9.1 ± 1.05	8.7 ± 2.36	**9.15**^**C**^
T_3_	9.8 ± 1.15	9.8 ± 1.49	9.8 ± 1.49	**9.72**^**A**^
**Mean**	**9.55**^**a**^	**9.23**^**c**^	**8.88** ^**d**^	**LSD = 0.68**
**Body & texture**				
Control	7.7 ± 0.57	7.7 ± 0.63	7.4 ± 0.63	**7.73**^**D**^
T_1_	8.6 ± 0.54	8.8 ± 0.52	8.8± 0.52	**8.63**^**C**^
T_2_	8.8 ± 0.49	9.1 ± 0.49	9.1 ± 0.48	**8.90**^**B**^
T_3_	9.4 ± 0.44	9.4 ± 0.44	9.4 ± 0.54	**9.27**^**A**^
**Mean**	**8.63**^**b**^	**8.75**^**a**^	**8.68**^**ab**^	**LSD = 0.36**
**Appearance & color**				
Control	9 ±1.05	9 ± 0.81	9 ± 1.05	**9.0** ^**A**^
T_1_	7 ± 0.62	7 ± 0.78	7 ± 0.97	**7.0**^**C**^
T_2_	8 ± 0.78	8 ± 1.02	8 ± 0.91	**8.0**^**B**^
T_3_	9 ± 0.91	9 ± 0.94	9 ± 0.82	**9.0**^**A**^
**Mean**	**8.25**^**a**^	**8.25**^**a**^	**8.25**^**a**^	**LSD = 0.39**
**Odour**				
Control	9 ± 0.94	9 ± 0.94	9 ±1.05	**9.0**^**A**^
T_1_	8 ± 1.129	8 ± 1.56	8 ± 1.50	**8.0**^**B**^
T_2_	9 ± 0.82	9 ± 0.82	9 ± 1.05	**9.0**^**A**^
T_3_	9 ± 0.82	9 ± 0.94	9 ± 0.82	**9.0**^**A**^
**Mean**	**8.75**^**a**^	**8.75**^**a**^	**8.75**^**a**^	**LSD = 0.47**
**Overall acceptability**				
Control	8.9 ± 2.35	8.0.± 1.7	8.4 ± 3.52	**8.73**^**C**^
T_1_	8.6 ± 2.6	8.5 ± 3.19	8.2 ± 82.24	**8.45**^**D**^
T_2_	9.1 ± 2.09	9.0 ± 1.96	8.8 ± 1.65	**8.95**^**B**^
T_3_	9.5 ± 2.55	9.5 ± 3.43	9.5 ± 2.21	**9.43**^**A**^
**Mean**	**9.04**^**a**^	**8.94**^**ab**^	**8.73**^**c**^	**LSD = 1.11**

C: Control plain yoghurt, T_1_; Yoghurt enriched with DPP grains, T_2_; Yoghurt enriched with DPP ethanol extract and T_3_; Yoghurt enriched with DPP nanoencapsulated extract (40%)

Data are expressed as means± standard deviation (n = 10).

Means with different superscripts in a row/column are significantly different at p<0.05.

## Conclusion

In conclusion, the date palm pollen evaluation revealed its rich content of protein, carbohydrate, minerals, unsaturated fatty acids ω-3, ω-6 and ω-9 and phenolic compounds (especially catechin) which was pronounced in its antioxidant potentials. Extraction of DPP in current study, increased the levels of unsaturated fatty acids (especially ω-9, ω-6 and ω-3) and decreased the saturated fatty acids content by almost two folds, in addition to raising the ω6/ ω3 ratio from 2.31 up to 2.83, it also elevated the antioxidant potentials which reflected on the yoghurt fortified products exerting functional properties with no effects on DPP functional groups as revealed in FTIR analyses or adverse effects on starter culture viability. Furthermore, fortifying with nanoencapsulated form enabled the reformation of yoghurt products microstructure to be more similar to control, enhanced the phase separation and consequently lowered the viscosity with no effects on product color. The DPP nanocapsules were proved to be safe as it did not show any adverse effects on RPE1 cells up to 100 μg/mL. The DPP fortified yoghurt types produced in the current study can be considered as symbiotic functional product as it contained both probiotics (10^6^ CFU/g) and prebiotics represented in DPP forms. The most ideal fortification form of the DPP three examined forms (DPP grains, DPP ethanol extract and DPP nanoencapsulated) which can be recommended was DPP nanoencapsulated form with all characteristics announced through microstructure, color, FTIR, physical, TPA, microbiological and sensorial analyses, providing new properties supporting their functional bioactive roles.

## Ethics statement

Sensory evaluation performed by testers at Food Technology Department, Arid Lands Cultivation Research Institute (ALCRI), City of Scientific Research and Technological Applications (SRTA-City), received waiver of the Institutional Review Board (IRB).

## References

[1] McKinley MC. The nutrition and health benefits of yoghurt. Int J Dairy Technol. 2005;58(1):1–12. 10.1111/j.1471-0307.2005.00180.x

[2] Lee WJ, Lucey JA. Formation and physical properties of yogurt. Asian-Australasian J Anim Sci. 2010;23(9):1127–36. 10.5713/ajas.2010.r.05

[3] Mehmood ST, Masud T, Mahmood T, Masqsud S. Effect of Different Additives from local source on the quality of yoghurt. Pakistan J Nutr. 2008;7(5):695–9. 10.3923/pjn.2008.695.699

[4] Kroyer G, Hegedus N. Evaluation of bioactive properties of pollen extracts as functional dietary food supplement. Innov Food Sci Emerg Technol. 2001;2(3):171–4. 10.1016/S1466-8564(01)00039-X

[5] Bishr M, Desoukey SY. Comparative Study of the Nutritional Value of Four Types of Egyptian Palm Pollens. J Pharm Nutr Sci. 2012;2(1):50–6. 10.6000/1927-5951.2012.02.01.7

[6] Vladimir-Knezevic S, Blazekovic MB, Stefan M, Babac M. Plant Polyphenols as Antioxidants Influencing the Human Health. In: Phytochemicals as Nutraceuticals—Global Approaches to Their Role in Nutrition and Health [Internet]. Dr Venkete. InTech; 2012 Available from: http://www.intechopen.com/books/phytochemicals-as-nutraceuticals-global-approaches-to-their-role-in-nutrition-and-health/plant-polyphenols-as-antioxidants-influencing-the-human-health. 10.5772/27843

[7] Aguiar J, Estevinho BN, Santos L. Microencapsulation of natural antioxidants for food application–The specific case of coffee antioxidants–A review. Trends Food Sci Technol [Internet]. 2016;58:21–39. Available from: 10.1016/j.tifs.2016.10.012

[8] de Souza Simões L, Madalena DA, Pinheiro AC, Teixeira JA, Vicente AA, Ramos ÓL. Micro- and nano bio-based delivery systems for food applications: In vitro behavior. Adv Colloid Interface Sci. 2017;243:23–45. 10.1016/j.cis.2017.02.010 28395856

[9] Rashidinejad A, Birch EJ, Sun-Waterhouse D, Everett DW. Effects of catechin on the phenolic content and antioxidant properties of low-fat cheese. Int J Food Sci Technol. 2013;48(12):2448–55. 10.1111/ijfs.12234

[10] Haratifar S, Meckling KA, Corredig M. Antiproliferative activity of tea catechins associated with casein micelles, using HT29 colon cancer cells. J Dairy Sci [Internet]. 2014;97(2):672–8. Available from: http://linkinghub.elsevier.com/retrieve/pii/S002203021300859X 10.3168/jds.2013-7263 24359816

[11] Livney YD. Nanostructured delivery systems in food: Latest developments and potential future directions. Curr Opin Food Sci [Internet]. 2015;3:125–35. Available from: 10.1016/j.cofs.2015.06.010

[12] Katouzian I, Jafari SM. Nano-encapsulation as a promising approach for targeted delivery and controlled release of vitamins. Trends Food Sci Technol [Internet]. 2016;53:34–48. Available from: 10.1016/j.tifs.2016.05.002

[13] Estevinho BN, Rocha F, Santos L, Alves A. Microencapsulation with chitosan by spray drying for industry applications—A review. Trends Food Sci Technol. 2013;31:138–55. 10.1016/j.tifs.2013.04.001

[14] AOAC International. Official methods of analysis 20th ed Latimer GW, editor. AOAC International, Rockville, Maryland 20850–3250, USA; 2016.

[15] Dubois M, Gilles KA, Hamilton JK, Rebers PA, Smith F. Colorimetric method for determination of sugars and related substances. Anal Chem. 1956;28:350–6. 10.1021/ac60111a017

[16] Beaty RD, Kerber JD. Concepts, Instrumentation and Techniques in Atomic Absorption Spectrophotometry second Edi North. The Perkin-Elmer Corporation, Norwalk, CT, U.S.A; 1993.

[17] Ling ER. A Textbook of Dairy Chemistry 2nd ed Vol. Two Practi. Chapman and Hall LTD, London; 1945.

[18] Croci AN, Cioroiu B, Lazar D. HPLC evaluation of phenolic and polyphenolic acids from propolis. Farmacia. 2009;LVII(1):52–7. https://www.researchgate.net/publication/289758264

[19] El-Neweshy MS, El-Maddawy ZK, El-Sayed YS. Therapeutic effects of date palm (Phoenix dactylifera L.) pollen extract on cadmium-induced testicular toxicity. Andrologia. 2013;45(6):369–78. 10.1111/and.12025 22998418

[20] Al-Owaisi M, Al-Hadiwi N, Khan SA. GC-MS analysis, determination of total phenolics, flavonoid content and free radical scavenging activities of various crude extracts of Moringa peregrina (Forssk.) Fiori leaves. Asian Pac J Trop Biomed [Internet]. 2014;4(12):964–70. Available from: 10.1016/j.rser.2010.07.054\nhttp://www.sciencedirect.com/science/article/pii/S222116911530112X

[21] Vardhanabhuti B, Foegeding EA, McGuffey MK, Daubert CR, Swaisgood HE. Gelation properties of dispersions containing polymerized and native whey protein isolate. Food Hydrocoll. 2001;15(2):165–75. 10.1016/S0268-005X(00)00062-X

[22] Munir H, Shahid M, Anjum F, Mudgil D. Structural, thermal and rheological characterization of modified Dalbergia sissoo gum-A medicinal gum. Int J Biol Macromol [Internet]. 2016;84:236–45. Available from: 10.1016/j.ijbiomac.2015.12.001 26709145

[23] Cerqueira MA, Souza BWS, Simoes J, Teixeira JA, Domingues MRM, Coimbra MA, et al Structural and thermal characterization of galactomannans from non-conventional sources. Carbohydr Polym [Internet]. 2011;83(1):179–85. Available from: 10.1016/j.carbpol.2010.07.036

[24] Schumann K, Siekmann K. Soaps. In: IUllmann’s Encyclopedia of Industrial Chemistry. Weinheim: Wiley- VCH; 2000 p. 241–61.

[25] Singleton VL, Orthofer R, Lamuela-Raventos RM. Analysis of total phenols and other oxidation substrates and antioxidants by means of Folin–Ciocalteau reagent. Oxidants and antioxidants part A. Vol. 299, Methods in Enzymology. Academic Press; 1999 152–178 p. 10.1016/S0076-6879(99)99017-1

[26] Sakanaka S, Tachibana Y, Okada Y. Preparation and antioxidant properties of extracts of Japanese persimmon leaf tea (kakinoha-cha). Food Chem. 2005;9:569–75. 10.1016/j.foodchem.2004.03.013

[27] Brand-Williams Cuvelier ME, Berset C. Use of a Free Radical Method to Evaluate Antioxidant Activity. Food Sci Technol. 1995;28:25–30. 10.1016/S0023-6438(95)80008-5

[28] Mosmann T. Rapid colorimetric assay for cellular growth and survival: Application to proliferation and cytotoxicity assays. J Immunol Methods. 1983;65(1–2):55–63. 10.1016/0022-1759(83)90303-4 6606682

[29] Bassyouni FA, Abu-Baker SM, Mahmoud K, Moharam M, El-Nakkady SS, Abdel-Rehim M. Synthesis and biological evaluation of some new triazolo[1,5-a]quinoline derivatives as anticancer and antimicrobial agents. RSC Adv. 2014;4(46):24131–41.

[30] Tamime AY, Robenson RK. Tamime and Robenson’s Yoghurt Science and Technology 3rd ed Cambridge England: Woodhead Publishing Ltd and CRC Press LLC; 2007. 808 p. eBook ISBN: 9781845692612

[31] Hunter RS, Harold RW. The measurement of appearance [Internet]. 2nd Editio A Wiley Interscience Publication, John Wiley & Sons. Inc; 1976 421 p. Available from: http://physicstoday.scitation.org/doi/10.1063/1.3024412

[32] Akalın AS, Unal G, Dinkci N, Hayaloglu AA. Microstructural, textural, and sensory characteristics of probiotic yogurts fortified with sodium calcium caseinate or whey protein concentrate. J Dairy Sci [Internet]. 2012;95(7):3617–28. Available from: http://linkinghub.elsevier.com/retrieve/pii/S0022030212003426 10.3168/jds.2011-5297 22720919

[33] Isanga J, Zhang G. Production and evaluation of some physicochemical parameters of peanut milk yoghurt. LWT—Food Sci Technol [Internet]. 2009;42(6):1132–8. Available from: 10.1016/j.lwt.2009.01.014

[34] Bourne MC. Food Texture and Viscosity: Concept and Measurement 2nd ed Elsevier Science & Technology Books; 2002.

[35] Szczesniak AS. Classification of Textural Characteristics. J Food Sci. 1963;28(4):385–9. 10.1111/j.1365-2621.1963.tb00215.x

[36] Marth EH. Standard Methods for the Examination of Dairy Products. 14th editi American Public Health Association 1015 Eighteenth Street, N.W. Washington, DC 20036; 1978. 456 p.

[37] Darwish AMG, Khalifa RE, El Sohaimy SA. Functional properties of chia seed mucilage supplemented in low fat yoghurt. Alexandria Sci Exch J. 2018;39(3):450–9. 10.21608/ASEJAIQJSAE.2018.13882

[38] Senaka Ranadheera C, Evans CA, Adams MC, Baines SK. Probiotic viability and physico-chemical and sensory properties of plain and stirred fruit yogurts made from goat’s milk. Food Chem [Internet]. 2012;135(3):1411–8. Available from: 10.1016/j.foodchem.2012.06.025 22953874

[39] Ozturk B, Argin S, Ozilgen M, McClements DJ. Nanoemulsion delivery systems for oil-soluble vitamins: Influence of carrier oil type on lipid digestion and vitamin D3 bioaccessibility. Food Chem. 2015;187:499–506. 10.1016/j.foodchem.2015.04.065 25977056

[40] ISO 22935–3 | IDF 099–3: 2009 –Milk and milk products–Sensory analysis–Part 3: Guidance on a method for evaluation of compliance with product specifications for sensory properties by scoring. First edit. ISO and IDF 2009; 2009. 7 p.

[41] Hassan HMM. Chemical Composition and Nutritional Value of Palm Pollen Grains. Glob J Biotechnol Biochem. 2011;6(1):1–7.

[42] Rasekh A, Jashni HK, Rahmanian K, Jahromi AS. Effect of palm pollen on sperm parameters of infertile man. Pakistan J Biol Sci. 2015;18(4):196–9. 10.3923/pjbs.2015.196.199 26506651

[43] Abed El-Azim MHM, Yassin FA, Khalil SA, Amani A, El-Mesalamy MD. Hydrocarbons, fatty acids and biological activity of date palm pollen (phoenix dactylifera L.) growing in Egypt. IOSR J Pharm Biol Sci Ver I [Internet]. 2015;10(3):2319–7676. Available from: www.iosrjournals.org 10.9790/3008-10314651

[44] Daoud A, Malika D, Bakari S, Hfaiedh N, Mnafgui K, Kadri A, et al Assessment of polyphenol composition, antioxidant and antimicrobial properties of various extracts of Date Palm Pollen (DPP) from two Tunisian cultivars. Arab J Chem. 2015; 10.1016/j.arabjc.2015.07.014

[45] Grzesik M, Naparło K, Bartosz G, Sadowska-Bartosz I. Antioxidant properties of catechins: Comparison with other antioxidants. Food Chem. 2018;241(June 2017):480–92. 10.1016/j.foodchem.2017.08.117 28958556

[46] Lima CF, Carvalho F, Fernandes E, Bastos ML, Santos-Gomes PC, Fernandes-Ferreira M, et al Evaluation of toxic/protective effects of the essential oil of Salvia officinalis on freshly isolated rat hepatocytes. Toxicol In Vitro. 2004;18(4):457–65. 10.1016/j.tiv.2004.01.001 15130603

[47] Besbes S, Blecker C, Deroanne C, Drira NE, Attia H. Date seeds: Chemical composition and characteristic profiles of the lipid fraction. Food Chem. 2004;84(4):577–84. 10.1016/S0308-8146(03)00281-4

[48] Simopoulos AP. Omega-6 / Omega-3 Essential Fatty Acid Ratio and Chronic Diseases. 2004;20(1):77–90. 10.3181/0711-MR-311

[49] Simopoulos AP. The importance of the ratio of omega-6 / omega-3 essential fatty acids. 2002;56:365–79. 10.1016/S0753-3322(02)00253-6 12442909

[50] Pan K, Zhong Q, Baek SJ. Enhanced dispersibility and bioactivity of curcumin by encapsulation in casein nanocapsules. J Agric Food Chem. 2013;61(25):6036–43. 10.1021/jf400752a 23734864

[51] Rezaei A, Fathi M, Jafari SM. Nanoencapsulation of hydrophobic and low-soluble food bioactive compounds within different nanocarriers. Vol. 88, Food Hydrocolloids. Elsevier B.V.; 2019 146–162 p. 10.1016/j.foodhyd.2018.10.003

[52] Cho H, Lee HJ, Yu KS, Choi YM, Hwang KT. Characterisation and food application of curcumin bound to sodium caseinate–polysaccharide electrostatic complexes. Int J Food Sci Technol. 2017;52(8):1770–6. 10.1111/ijfs.13450

[53] Almehdi AM, Maraqa M, Abdulkhalik S. Aerobiological studies and low allerginicity of Date-Palm pollen in the UAE. Int J Environ Health Res. 2005;15(3):217–24. 10.1080/09603120500105745 16134484

[54] Jazinizadeh E, Majd A, Pourpak Z. Anther development and microsporogenesis in date palm (Phoenix dactylifera L.) 2017;49(1):331–5.

[55] Oladoja NA, Unuabonah EI, Amuda OS, Kolawole OM. Polysaccharides as a green and sustainable resources for water and wastewater treatment [Internet]. Cham, Switzerland: Springer Nature; 2017 Available from: http://link.springer.com/10.1007/978-3-319-56599-6

[56] Farouk A, Metwaly A, Mohsen M. Chemical Composition and antioxidant activity of Date Palm pollen grains (Phoenix dactylifera L. Palmae) essential oil for Siwe Cultivar Cultivated in Egypt. Middle East J Appl Sci. 2015;5(4):945–9. https://www.researchgate.net/publication/320290100

[57] Andersen FA. Final report on the safety assessment of lecithin and hydrogenated lecithin. Int J Toxicol. 2001;21(SUPPL. 2):21–45. 10.1080/109158101750300937 11358109

[58] Jahromi AR, Jashni HK, Rahmanian K, Jahromi AS. Effect of palm pollen on sperm parameters of infertile man. Pakistan J Biol Sci. 2015;18(4):196–9. 10.3923/pjbs.2015.196.199 26506651

[59] Metry WA, Owayss AA. Influence of incorporating honey and royal jelly on the quality of yoghurt during storage. Egypt J Food Sci. 2009;37:115–31. https://www.researchgate.net/publication/283257408

[60] Yerlikaya O. Effect of bee pollen supplement on antimicrobial, chemical, rheological, sensorial properties and probiotic viability of fermented milk beverages. Mljekarstvo [Internet]. 2014;64(4):268–79. Available from: http://hrcak.srce.hr/index.php?show=clanak&id_clanak_jezik=191970 10.15567/mljekarstvo.2014.0406

[61] Pichot R, Watson RL, Norton IT. Phospholipids at the interface: Current trends and challenges. Int J Mol Sci. 2013;14(6):11767–94. 10.3390/ijms140611767 23736688 PMC3709755

[62] Puvanenthiran A, Williams RPW, Augustin MA. Structure and visco-elastic properties of set yoghurt with altered casein to whey protein ratios. Int Dairy J. 2002;12(4):383–91. 10.1016/S0958-6946(02)00033-X

[63] Lucey JA. Formation and physical properties of milk protein gels. J Dairy Sci [Internet]. 2002;85(2):281–94. Available from: http://linkinghub.elsevier.com/retrieve/pii/S0022030202740782 10.3168/jds.S0022-0302(02)74078- 11913691

[64] Coggins PC, Rowe DE, Wilson JC, Kumari S. Storage and temperature effects on appearance and textural characteristics of conventional milk yogurt. J Sens Stud. 2010;25(4):549–76. 10.1111/j.1745-459X.2010.00286.x

[65] de Souza Oliveira RP, Perego P, de Oliveira MN, Converti A. Effect of inulin on the growth and metabolism of a probiotic strain of Lactobacillus rhamnosus in co-culture with Streptococcus thermophilus. LWT—Food Sci Technol [Internet]. 2012;47(2):358–63. Available from: 10.1016/j.lwt.2012.01.031

[66] El-Kadi SL, Ismail MM, Zidan MS. Chemical and Microbial Characterizations of Bio-Yoghurt Made Using ABT Culture, Cow Milk and Coconut Milk. Ec Microbiol. 2017;5(3):109–24.

